# Milk Diet in the Puerperal State

**Published:** 1878-10-20

**Authors:** 


### MILK DIET IN THE PUERPERAL STATE.

Dr. S. G. Hardman writes—

HARMONY GROVE, GA., Oct. 4th, 1878.

*Editor Souiheru Med. Record:*

As I find some yet in darkness on the subject of sweet milk in the puerperal state, I would be glad to have you notice this in your most excellent Journal. Whether milk is or is not a proper diet for a woman in the puerperal state.

We do not believe that there is any special reason for the rejection of milk from the list of dietetic articles allowed the puerperal woman. It is usual and best in early child-bed, say for the first six or eight days, to rely upon toast, broths, tea and such articles of food as are light and unirritating, by reason of the feverish tendency usual to this condition. Milk is a rich article of diet, and has been found frequently to disagree with adult stomachs, tending to constipation and flatulence, for which reason we have been accustomed to exclude it, and not because we believe there exists any special physiological contra-indication to its use in the puerperal state.—[Ed. Rec.

